# Relationship between Pyruvate Kinase Activity and Cariogenic Biofilm Formation in *Streptococcus mutans* Biotypes in Caries Patients

**DOI:** 10.3389/fmicb.2017.00856

**Published:** 2017-05-16

**Authors:** Wirginia Krzyściak, Monika Papież, Anna Jurczak, Dorota Kościelniak, Palina Vyhouskaya, Katarzyna Zagórska-Świeży, Anna Skalniak

**Affiliations:** ^1^Department of Medical Diagnostics, Faculty of Pharmacy, Jagiellonian University Medical CollegeKrakow, Poland; ^2^Department of Cytobiology, Faculty of Pharmacy, Jagiellonian University Medical CollegeKrakow, Poland; ^3^Department of Pediatric Dentistry, Institute of Dentistry, Jagiellonian University Medical CollegeKrakow, Poland; ^4^Department of Otorhinolaryngology, Jagiellonian University Medical CollegeKrakow, Poland; ^5^Genetics Laboratory, Department of Endocrinology, Jagiellonian University Medical CollegeKrakow, Poland

**Keywords:** *Streptococcus mutans*, pyruvate kinase, biofilm, caries, biotypes

## Abstract

*Streptococcus mutans* (MS) and its biotype I are the strains most frequently found in dental plaque of young children. Our results indicate that in children pyruvate kinase (PK) activity increases significantly in dental plaque, and this corresponds with caries progression. The MS strains isolated in this study or their main glycolytic metabolism connected with PK enzymes might be useful risk factors for studying the pathogenesis and target points of novel therapies for dental caries. The relationship between PK activity, cariogenic biofilm formation and selected biotypes occurrence was studied. *S. mutans* dental plaque samples were collected from supragingival plaque of individual deciduous molars in 143 subjects. PK activity was measured at different time points during biofilm formation. Patients were divided into two groups: initial stage decay, and extensive decay. Non-parametric analysis of variance and analysis of covariance were used to determine the connections between *S. mutans* levels, PK activity and dental caries biotypes. A total of 143 strains were derived from subjects with caries. Biotyping data showed that 62, 23, 50, and 8 strains were classified as biotypes I, II, III, IV, respectively. PK activity in biotypes I, II, and IV was significantly higher in comparison to that in biotype III. The correlation between the level of *S. mutans* in dental plaque and PK activity was both statistically significant (*p* < 0.05) and positive. The greater the level of *S. mutans* in the biofilm (colony count and total biomass), the higher the PK activity; similarly, a low bacterial count correlated with low PK activity.

## Introduction

The ability to form biofilm is the main determinant of the pathogenicity of bacterial strains that cause dental caries. Although bacterial plaque-forming flora is very complex and heterogeneous, *Streptococcus mutans* is one of the major etiologic factors of caries (Jiang et al., 2016).

*Streptococcus mutans* has a number of features that under appropriate environmental conditions determine its pathogenicity. One of its main determinants enabling *S. mutans* to colonize and survive in the oral cavity is the ability to generate adhesion and biofilm formation (Demonte et al., 2017). In the conditions of the oral cavity, the cariogenic bacteria *S. mutans* are characterized by altered metabolism compared to cells of normal flora. It is based on glycolysis, which also occurs in the presence of oxygen. Low oxygen concentration (<2%), i.e., hypoxia occurring inside the biofilm, increases the expression of genes encoding glycolytic enzymes and inhibits oxidative phosphorylation. Pyruvate kinase (PK) is one of the key enzymes involved in this process and catalyzes its last step: the transfer of a phosphate group from phosphoenolpyruvate (PEP) to ADP giving pyruvate and ATP. This reaction is considered to determine the speed of the entire glycolysis in *S. mutans*.

Available literature data suggest different methods of typing *S. mutans* strains; some of these are based on the antigenic heterogeneity of cell wall carbohydrates (De Soet and De Graaff, 1990; Nakano et al., 2005), while others detect differences in sensitivity to selected antibiotics (Palmer et al., 2013; Liu et al., 2016), or in the production of bacteriocins (Tahmourespour et al., 2014; Momeni et al., 2015). Ribotyping based on the genes encoding ribosomal RNA has also been widely reported (Zhan et al., 2012).

Typing of viridans group streptococci (VGS) based on enzymatic reactions was first introduced by Facklam (1977). That study included 1227 clinical and 80 stock strains which were differentiated (97% of the strains) into 10 species with the use of 25 biochemical tests. This scheme became the basis for modern commercial identification tests such as STREPTOtest 24 (Pliva-Lachema) and API 20 Strep (bioMerieux) (Menon, 2016). Later, Facklam suggested a VGS biotyping scheme based on six reactions (production of acetoin, hydrolysis of aesculin, arginine and urea as well as fermentation of mannitol and sorbitol) (Facklam, 2002).

Biotyping using the activity of phosphatase inulin, tegatose and melibiose was applied and it was demonstrated that among four obtained biotypes (three using clusterization method), biotype I was predominant (64%), followed by biotypes II (26%), III (6%), and IV (4%) (Krzyściak et al., 2014). Compatible results (where biotype I, or *c* was dominant) were obtained by Hofling et al. (1998) (*S. mutans* constituted 78%), Oldershaw et al. (1982), and Farghaly et al. (1984).

De La Higuera et al. (1999) performed a differentiation of 160 *S. mutans* isolates using an API ZYM commercial enzymatic kit (bioMérieux). Three enzymes were used (valine aryl amidase, acid phosphatase, and α-galactosidase); additionally, susceptibility tests to amoxycillin, cefazolin, erythromycin, clindamycin, vancomycin, teicoplanin, and imipenem were performed. Biotype V (valine arylamidase negative, acid phosphatase, and α-galactosidase positive) was found to be the most prevalent (31.25%).

Regarding more recent research, Ravindran et al. (2013) applied biochemical tests, i.e., hydrolysis of arginine, fermentation of sugars (mannitol, sorbitol, inulin, melibiose, and raffinose), as well as bacitracin resistance and aerobic growth. Biotype I was the most common in both *S. mutans* and *S. sobrinus* species (40.4 and 60%, respectively). Yoo et al. (2007) performed biotyping of 95 strains of MS based on reactions of sugar fermentation (mannitol, melibiose, raffinose, sorbitol), and arginine degradation. Apart from several atypical strain profiles, frequencies of particular biotypes were: I – 65.26%, II – 1.05%, IV – 2.11%, V – 10.53%.

A comparison of several biotyping methods (biochemical kits En-coccus test, Pliva-LaChema; API20 Strep, bioMerieux; genetic methods *ddl*-PCR and ITS-PCR) was performed by Brtkova et al. (2010). Collected samples contained animal enterococci and results of both biochemical and genetic tests were consistent. Teles et al. (2011) tested VGS with commercial kits: API^®^ Rapid 32 ID Strep (bioMérieux) and Vitek^®^ 2 (bioMérieux). The API test appeared to better identify tested microorganisms (79% species had adequate results compared to 55% with the Vitek test). Some differences observed between particular kits are a result of different discrimination rates due to, e.g., a wider panel of tested compounds/enzymes (for example, API20 Strep 20 enables higher discrimination in comparison to En-coccus – 20 and 8 tested saccharides, respectively) (Brtkova et al., 2010).

Biotyping based on differences in the activity of selected enzymes from commercial tests used in diagnosis seems to be a good alternative to genotyping methods which, although they have become a quality standard in epidemiological studies in many countries in the world, are still not available for routine use in diagnostic procedures in small medical units, and in developing countries with limited financial resources allocated for health care. In addition, enzymatic typing is particularly difficult for *S. mutans*. This is because most biochemical processes associated with the metabolism of the above-mentioned bacteria appear to be limited and difficult to interpret by differentiating strains in order to assign species to their corresponding biotypes. Mistakes in interpretation can also result from the fact that the term “biotype of *S. mutans*” can be used in relation to a subspecies of the mutans group pre-defined in its nomenclature and classified on the basis of one genotype, phenotype, or serotype; e.g., recognizing bacteria belonging to biotype I not as a subtype within the same species but as a distinct species.

The proper typing of cariogenic bacterial strains may throw new light on their origin and prevalence in the population and thus increase the coverage of prevention activities in the field of tooth decay in children, especially in areas with an increased incidence of this disease.

The aim of this study was the determination of PK activity in *S. mutans* during biofilm formation and an assessment of the relationship of PK with selected biotypes for 143 strains of *S. mutans* isolated from dental plaque of children with varying severity of dental caries.

## Materials and Methods

### Study Groups

The study was performed in accordance with the Helsinki Declaration of 2013. Informed consent to the study procedure was obtained from all participants. The protocol was approved by the Bioethics Committee at the Jagiellonian University in Krakow (No. 122.6120.99.2016). Informed consent was obtained from all individual parents.

The study was conducted between 2015 and 2016 and included 143 pediatric patients from the Department of Pediatric Dentistry, Institute of Dentistry, Jagiellonian University in Krakow, Poland. The study included bacterial strains isolated from dental plaque of patients (*n* = 143; average age 4.6 ± 0.76) diagnosed with early childhood caries (ECC) of the deciduous teeth. The results marked with reference numbers were entered onto a standardized examination chart. Examinations were conducted based on the criteria established by the World Health Organization for epidemiological studies (WHO, 1997).

Children were divided into two subgroups: subjects with initial stage decay, defined as the non-cavitated group (1–2 in ICDAS codes); and subjects with extensive decay, defined as the cavitated group (5–6 in ICDAS II codes) (International Caries Detection and Assessment System Coordinating Committee, 2012). Ninety-seven patients were qualified to the non-cavitated group (47 girls, aged 4.68 ± 0.91 years and 50 boys, aged 4.6 ± 0.61). Forty six patients were assigned to the cavitated group (20 girls, aged 4.75 ± 0.85 years and 26 boys, aged 4.35 ± 0.63).

The exclusion criteria were as follows: age below 2 or above 6 years, diabetes, periodontal disease, epithelial dysplasia, and inflammatory lesions of the oral mucosa. Antibiotics, non-steroid, anti-inflammatory medications, corticosteroids, and vitamin intake within the last 3 months also resulted in a patient’s exclusion from the study. Dental plaque was determined using the OHI-S index (Simplified Oral Hygiene Index) (Wei and Lang, 1982).

### Dental Plaque Collection

After classification of the patients for the research, supragingival plaque (pooled from all surfaces of individual primary first teeth) samples were collected using sterile dental explorers. Each subject rinsed their mouth for 1 min with distilled water, before plaque collection. Material was collected from fasted participants in the morning (between 8 AM and 10 AM), before toothbrushing and any clinical examination. The collected plaque was put into sterile tubes with 0.5 ml phosphate buffered saline (PBS), inhibiting O_2_, and carried on ice to the lab within 1 h. Plaque samples were then sonicated for 30 s, vortexed to disperse and to obtain a homogeneous suspension and clarified by centrifugation at 2000 × *g* for 10 min at 4°C. A total of 50 μl aliquots of clarified supernatants were used for traditional plate culturing methods on HLR-S medium [HL Ritz medium containing 40 g tryptic soy agar (TSA), 20% sucrose, 0.3 U/ml bacitracin, 1.75 μg/ml polymyxin B sulfate and 0.5 μg/ml crystal violet] for three 10-fold dilutions of each plaque sample. All plates were incubated under 85% N_2_, 10% CO_2_, 5% O_2_ conditions at 37°C for 48 h. Samples with colony counts of more than 130 (10^4^ cells/ml) were considered positive.

### Morphological Identification

After obtaining pure colony samples on the HLR-S medium, single colonies were inoculated on the surface of TSA plates with 5% sheep blood and incubated under previously mentioned conditions. Then, the characteristics of individual colonies were evaluated, including the colonial shape and form, the type of hemolysis, and the Gram-staining.

### Bacterial Identification – Biotyping

Phenotypic identification of the isolated *S. mutans* species was performed using a commercial biochemistry identification test: STREPTOtest24 (Lachema, Pliva).

Biotyping from selected enzymatic reactions of the above commercial test was performed for *S. mutans* as the dominant bacteria species. Two approaches to biotyping criteria based on enzymatic activity profiles were used. The first approach consisted of the selection of appropriate enzymes (INU, MLB, TGT) based on the analysis of their activity in the population of the tested strains.

The second method for biotype determination consisted of the use of an unattended (without *a priori* available knowledge) statistical data analysis. Data were divided into groups (clusters), so that each group was as homogenous as possible (strains within the group were similar) and at the same time, clusters differed from one another (strains from different groups had the lowest possible number of common characters).

### Preparation of *S. mutans* Inoculum

Single pure *S. mutans* colonies were grown in 4 ml of BHI broth (Difco, BD, USA) supplemented with 5% sucrose for 8 h. Cells were harvested in the logarithmic phase of growth and washed three times with 40 mM potassium phosphate buffer (pH 7.0).

In the growth of *S. mutans* were analyzed by using a flow cytometer (LSRII, BD Biosciences Immunocytometry Systems, San Jose, CA, USA). Aggregates and doublets were excluded using a gating strategy related to the width versus height of the forward scatter (FSC) and side scatter (SSC).

A standardized suspension of the tested microorganisms was prepared in PBS to give 1 on the McFarland scale using a MicroSpek dual DSM densitometer. The density of the inoculum [1 × 10^7^ colony forming units (CFUs)/ml] was confirmed by counting single colonies after 24 h growth under the same conditions as described for BHI agar for *S. mutans*.

### *In Vitro* Biofilm Formation

The capacity for biofilm formation was evaluated using a closed model based on a microtiter plate. The biomass of molded/non-degraded biofilm was measured by staining with crystal violet (CV) according to the method described by Peeters et al. (2008). At the outset, 100 μl of a standardized *S. mutans* suspension was transferred to each well of a 96-well microtiter plate, which was incubated for 90 min to start the initial adhesion of *S. mutans*. The suspension was aspirated and each well was washed three times with PBS to remove planktonic cells. Subsequently, 100 μl of fetal bovine serum was added to each well and incubated under the same conditions for 8 h. The wells were washed three times in PBS and 100 μl of a standardized *S. mutans* suspension was added to each well. Control samples were 100 μl of PBS (PBS control) or 100 ml of BHI broth with 5% sucrose (medium control).

To initiate biofilm growth and its further development, 60 μl of BHI with 5% sucrose was added to each well. The medium was changed once and the plates were incubated for 48 h.

### Analysis of Biofilm Formation by Determining CFU/ml

After 4, 6, 8, 10, 12, 14, 16, 18, 20, 22, and 24 h of incubation, the cells were washed twice with PBS. After addition of PBS, the biofilm was broken by homogenization in an ultrasonic homogenizer (Hielscher UP50H) for 30 s at an amplitude of 25%. Subsequently, serial dilutions of the solution were prepared by inoculating MSBS medium (containing bacitracin and sucrose) with 100 μl of the bacterial suspension and incubated for 48 h. The number of colonies (CFU/ml) was determined. The test was repeated in the two experiments at different time points, with a number of repeats in groups.

### Analysis of Biofilm Formation by Determining Total Biomass

Biofilm biomass was evaluated after 4, 6, 8, 10, 12, 14, 16, 18, and 24 h of incubation by staining with (CV). The biofilm was maintained in 99% methanol for 20 min, and air dried. Subsequently, 125 μl of 0.1% CV solution was added for 20 min, and the wells were rinsed three times with PBS. After each washing step, plates were dried by shaking. After the last washing step, the samples were allowed to dry thoroughly. Finally, bounded CV was released by adding 200 μl of 95% ethanol, followed by incubation for 15 min at room temperature. The contents of the wells were mixed by repeated pipetting and then 125 μl of the suspension was transferred to the wells of a clean 96-well flat-bottom microtiter plate. The absorbance was measured at a wavelength of λ_max_ = 540 nm. All steps were performed at room temperature. The study was conducted in two independent experiments. A biofilm formation curve was plotted based on the obtained data.

### Analysis of Biofilm Formation by Scanning Electron Microscopy (SEM)

Cover glasses (Agar Scientific, UK) of a 13 mm diameter were placed in the wells of a 24-well plate for biofilm formation in accordance with the procedure described above. At 4, 6, 8, 10, 12, 14, 16, 18, 20, 22, and 24 h, the samples were fixed in 1 ml of 2.5% glutaraldehyde solution for 1 h, dehydrated in a graded ethanol series for 20 min each, followed by immersion in 100% alcohol for 1 h. The disks were dried in an incubator for 24 h. After drying, the sample was transferred into aluminum sections and sprayed with gold (160 s, 40 mA). The samples were examined and photographed under a JEOL JSM-35CF scanning electron microscope (SEM) at 20–25 kV in the SEM Laboratory of the Otolaryngology Clinic, University Hospital in Krakow, Poland. These experiments were performed at each time point on three cultures of mono-species biofilm.

### Measurement of Pyruvate Kinase Activity

Pyruvate kinase activity was determined using *S. mutans* cells after 4, 6, 8, 10, 12, 14, 16, 18, 20, 22, and 24 h of a single-species biofilm formation using a continuous assay coupled with lactate dehydrogenase (LDH), in which the change in absorbance at 340 nm due to oxidation of NADH was measured using a POLARstar Omega microplate spectrophotometer (BMG Labtech, Germany).

Obtained cells were inoculated into 396 ml of sterile 40 mM potassium phosphate buffer (pH = 7.0) containing 10 mM beta-mercaptoethanol (BME), and grown for 24 h. Subsequently, cells were disrupted by sonic oscillation (15 min, 0°C, 200 W, 2A, Hielscher UP50H oscillator). The cell extract was centrifuged (17,500 × *g* for 30 min at 4°C) and the supernatant was used for further purification of the enzyme. All subsequent procedures were performed on ice.

Cleaning efficiency is ∼140-fold and recovery amounted to ∼10%. The purified enzyme was stored at -20°C in TRIS buffer with 10% glycerol until further enzymatic activity analysis.

The reaction for PK activity measurement contained 1 mM ADP, 1 mM phosphoenolpyruvate (PEP), 0.4 mM glucose-6-phosphate (G6P), 0.12 mM NADH (Santa Cruz Biotechnology, USA),10 μg/ml LDH (Carl Roth, Germany), 100 mM KCl, 10 mM MgCl_2_, and 0.13 μg/ml of the tested sample containing PK in 67 mM Tris-HCl buffer (pH 7.0) in a total volume of 250 μl. The activity was determined spectrophotometrically by recording the change in absorbance at 340 nm (μmol/min/mg of protein). PK activity was defined as the quantity of PK which catalyzed the formation of one micromole of PEP per minute. The reaction was carried out for 10 min at 30°C on a POLARstar Omega microplate spectrophotometer (BMG Labtech, Germany). Values of kinetic parameters such as *K_m_* and *V_max_* were calculated with respect to the change in the substrate (PEP) concentration (0–2.0 mM) at a fixed ADP concentration of 1 mM fitting in GraphPad Prism 7 (GraphPad TM Software, USA).

### Statistical Analysis

Statistical analysis was performed using R 3.2.3 (R Development Core Team, 2009). The normality of the data was assessed by the Shapiro–Wilk test and, where appropriate, non-parametric analyses were performed. Data are expressed as median ± upper and lower quartiles. Strains were divided into clusters using agglomerative hierarchical clustering approach with common setting: complete linkage and Ward metric. To determine the relationship between PK, CFU, and the optical density (OD) and the stage of disease and biotypes, the Kruskal–Wallis test was used. To answer the question as to how exactly this dependence looks in particular biotypes, a *post hoc* analysis was made (Dunn’s test). Markers were ascertained using the Spearman coefficient of correlation, analysis of covariance (ANCOVA) was performed to assess the connections between PK, CFU and OD, biotypes and the forms of the disease, corrected for the effect of confounding factors (age, sex). A value of *p* < 0.05 was considered statistically significant.

## Results

### Biotyping

In this project a total of 143 strains of *S. mutans* were isolated from supragingival plaque. Two approaches were used in determining the biotyping criteria based on enzymatic activity profiles of STREPTOtest 24.

The first approach was to select appropriate enzymes based on an analysis of their activity in the tested strain population. Three enzymes were selected, whose incidence among the tested strains was in a quartile deviation between 15 and 85%. These are inulin – 40.56% melibiose – 83.22%, and tagatose – 21.68%. The other 21 enzymes occurring in the test (STREPTOtest24) were rejected, because they did not meet the established criteria for enzyme inclusion (i.e., a frequency or enzyme activity between 15 and 85% in the test population).

The acceptance of these enzymes for biotyping criteria allowed the definition of eight enzymatic profiles, conventionally denoted by consecutive letters of the alphabet from A to H, as consistently shown: profile A occurring in 0.70% of the strains was characterized by a lack of activity of inulin (INU), melibiose (MLB), and tagatose (TGT). Profile B occurring in 6.99% of the strains showed tagatose activity and inactive inulin and melibiose. Profile C occurring in 42.66% of the strains was characterized by melibiose activity and inactive inulin and tagatose. Profile D with inactive inulin and active melibiose and tagatose occurred in 9.09% of the strains. Profile E with inactive melibiose and tagatose, and active inulin occurred in 7.69% of the strains. Profile F was characterized by a lack of melibiose activity, active inulin and tagatose, and occurred in 1.40% of the strains. Profile G was characterized by a lack of tagatose activity, active inulin and melibiose and occurred in 27.27% of the strains. Profile H was characterized by the activity of all three enzymes (inulin, melibiose, and tagatose) and occurred in less than 4.20% of the strains. The most numerously represented profile is ‘C,’ characterized by a lack of inulin or tagatose activity.

The distribution of the obtained profiles was heterogeneous; therefore, strains whose profiles differed only according to MLB were grouped into one biotype (MLB was the nearest to being excluded from the range 15–85%). The following four biotypes are given in four Roman numerals: I–IV. Biotype I, with a frequency of 43.36% (*n* = 62), contained profiles A and C, and showed melibiose activity without active inulin and tagatose. Biotype II (16.08%, *n* = 23) consisted of profiles B and D and showed tagatose activity, and inactive inulin with partially active melibiose. Biotype III (34.97%, *n* = 50) consisted of profiles E and G, and included strains with active inulin, inactive tagatose and partial melibiose activity. Biotype IV (5.59%, *n* = 8) consisted of profiles F and H and was characterized by partial melibiose activity and active inulin and tagatose.

Another way to determine biotypes was the application of statistical unattended (without *a priori* available knowledge) data analysis.

**FIGURE 1:**
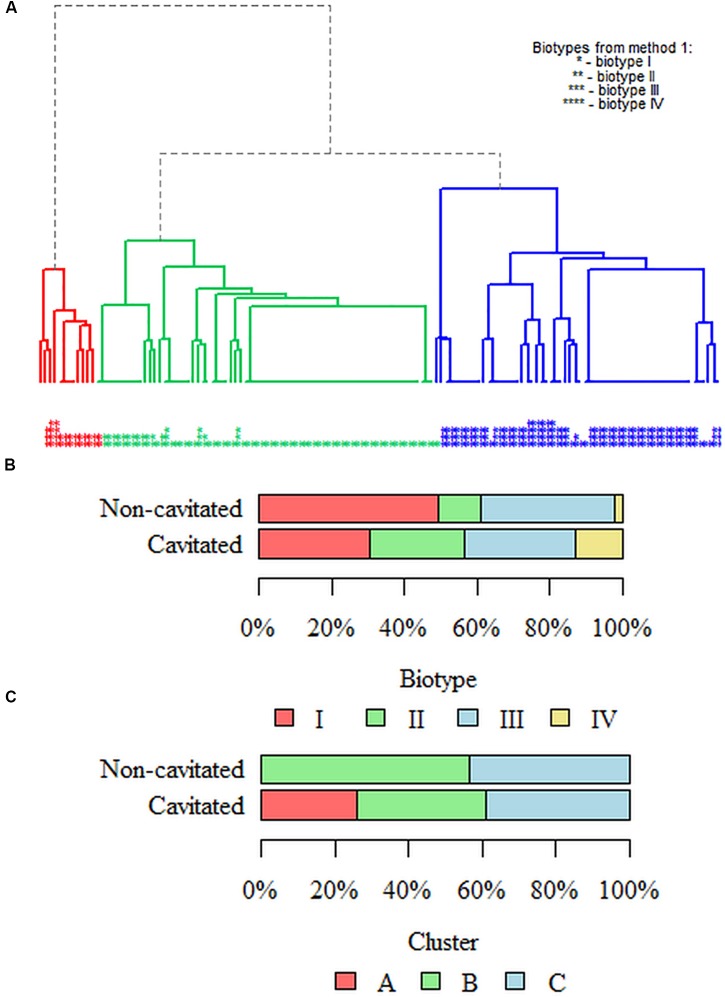
**Dendrogram with three marked biotypes (clusters) of the studied *Streptococcus mutans* population**. The criterion for enzyme biotyping in the arbitrary method was the selection of chosen enzymes based on the analysis of their activity in the population of the tested strains. We have included those enzymes whose activity in the test population is in the 15–85% quartile deviation. Hence, there are three enzymes: inulin, melibiose and tagatose, giving eight profiles eventually resulting in four biotypes (I–IV) marked on the chart with stars (^∗^–^∗∗∗∗^). Three clusters [the main branches in the figure colored red (cluster **A**), green (cluster **B**) and blue (cluster **C**)] were obtained by clustering using the hierarchical method. In the unsupervised clusterization method (without *a priori* knowledge), data were divided into clusters so that each group was as homogeneous as possible (the strains within the groups are similar), and the clusters were mutually different (strains from different groups have as few common features as possible). For the best-fitted dendrogram, strains were differentiated on the basis of inulin, melibiose and tagatose activity. This allowed for the differentiation of three clusters – the main branches in the figure in different colors and described in the text as **A–C**. The distribution of enzymatic profiles obtained by the arbitrary method (method no. 1) is also shown. The red cluster **(A)** is basically biotype II; green is biotype I, and blue is biotype III.

The results of the division into clusters are shown in dendrograms. Strains were differentiated based on the activity of melibiose and tagatose, which enabled the extraction of three biotypes – the main branches of the diagram (**Figure 1**) marked with the colors red, green and blue as A, B, C. The figure also indicates the distribution of enzymatic profiles obtained using the arbitrary method; red cluster is biotype II; green is biotype I, and blue is biotype III.

### Biotypes in Caries Stages

While performing analysis in terms of the severity of the decay of four biotypes obtained in the arbitrary method, it was noted that the analyzed groups differed significantly (*p* = 0.003; Fisher’s exact test) (**Figures 1A,B**). The strains belonging to biotype I in the non-cavitated group constituted 49.48% (*n* = 48) and 30.43% (*n* = 14) in the cavitated group. The strains classified to biotype II constituted 11.34% (*n* = 11) in the non-cavitated group and 26.09% (*n* = 12) in the cavitated one. In turn, strains from biotype III were 37.11% (*n* = 36) in the non-cavitated and 30.43% (*n* = 14) in the cavitated group. In the case of biotype IV, strains from the non-cavitated group were less than 2.06% (*n* = 2) and 13.04% (*n* = 6) in the cavitated group.

While performing clustering in terms of the severity of decay of three clusters from the most well-fitted dendrogram, it was noted that the analyzed groups differed significantly (*p* < 0.001; Fisher’s exact test) (**Figures 1A,C**). Strains classified in cluster A in the non-cavitated group represented 0% (*n* = 0) and 26.09% (*n* = 12) in the cavitated group. In cluster B, 56.70% (*n* = 55) of the strains were non-cavitated and 34.78% (*n* = 16) belonged to the cavitated group. In cluster C, 43.30% (*n* = 42) of the strains belonged to the non-cavitated group while 39.13% (*n* = 18) to the cavitated one.

### Analysis of Biofilm Formation by Determining CFU/ml in Early Childhood Caries Stages

Logs of the number of microorganisms (*S. mutans*) (CFU/ml) forming a biofilm at the respective time points are statistically significant between both groups. *p*-value is less than 0.05 for all time points (4–24 h); therefore, the analyzed groups differed significantly in terms of the amount of microorganisms at any time point. At each of the time points of single-species biofilm formation, the amount of *S. mutans* in the –cavitated– group was significantly higher than in the –non-cavitated– one.

The highest number of microorganisms forming a biofilm was observed after 18 h in strains isolated from dental plaque from the cavitated group of children compared with non-cavitated children (7.67 ± 7.65–7.69 vs. 7.54 ± 7.52–7.57, *p* < 0.001; *U* Mann–Whitney test).

The number of microorganisms (*S. mutans*) (log CFU/ml) forming a biofilm at the respective time points was not dependent on sex (*p* > 0.05 for all time points, *U* Mann–Whitney test).

### Analysis of Biofilm Formation by Determining Total Biomass in Early Childhood Caries Stages

The total biomasses of microorganisms (*S. mutans*) forming a biofilm at the respective time points are statistically significant. *p*-value is less than 0.05 for all time points (4–24 h); therefore, the analyzed groups differed significantly in terms of the amount of microorganisms at any time point. At each of the time points of single-species biofilm formation, the amount of *S. mutan*s in the cavitated group was significantly higher than in the non-cavitated one.

The highest absorbance (the greatest amount of microorganisms cumulated in the biofilm) was observed after 18 h in strains isolated from dental plaque from the cavitated group of children compared with non-cavitated children (0.13 vs. 0.12, *p* < 0.001; *U* Mann–Whitney test).

The total biomass of microorganisms (*S. mutans*) forming biofilm at the particular time points did not depend on sex (*p* > 0.05 for all time points; *U* Mann–Whitney test).

**FIGURE 2:**
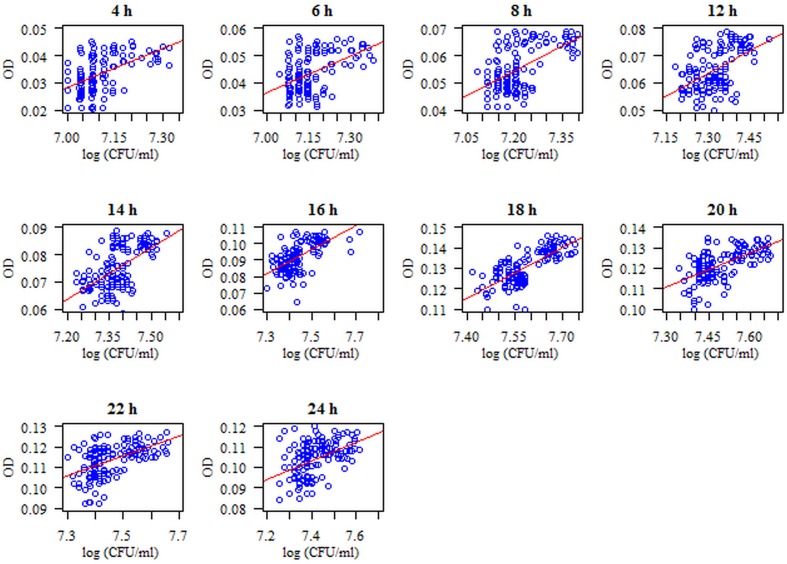
**Diagram of the amount of biofilm-forming microorganisms (*S. mutans*) (log CFU/ml) with respect to microbial biomass (*S. mutans*) using the method of crystal violet (CV) staining at particular time points of single-species biofilm formation**. The analyzed features had no normal distribution (*p* < 0.05, Shapiro–Wilk test), so a Spearman correlation coefficient was used for the analysis. The correlation between the amount of microorganisms expressed as log CFU/ml and the biomass of the formed biofilm expressed as OD (optical density measured at a wavelength λ_max_ = 540 nm) is statistically significant at each time point (*p* < 0.05). These dependencies are positive, i.e., the higher the log CFU/ml, the higher the OD and *vice versa.*

Attention was paid to the relationship between the number of biofilm-forming microorganisms (*S. mutans*) (log CFU/ml) and their biomass (OD) at the respective time points. The correlation is statistically significant (*p* < 0.05) at each time point of biofilm formation. These relationships are positive: the higher the log (CFU/ml), the higher the OD, and *vice versa*. The strongest relationship was noted after 18 h of biofilm formation (coefficient of correlation *r* = 0.71, *p* < 0.001) (**Figure 2**).

**FIGURE 3:**
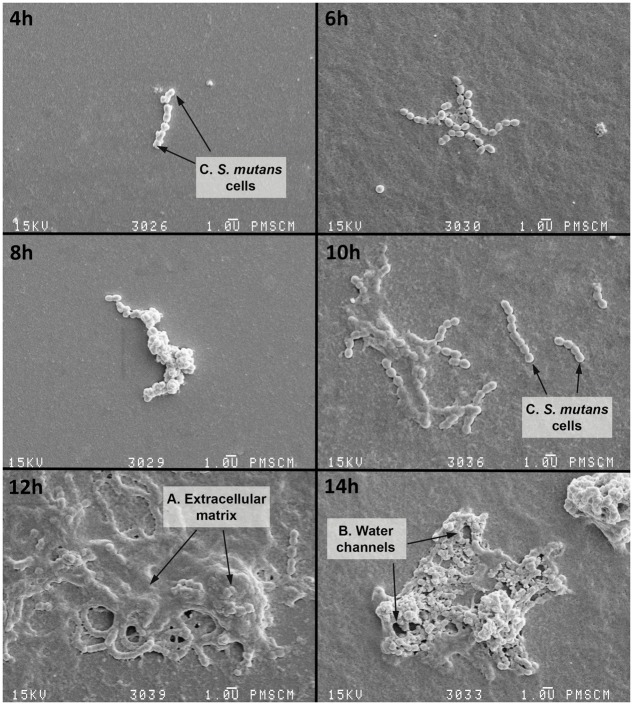
**Scanning electron microscopy (SEM) of the single-species biofilm formed by *S. mutans* cells at various time points**. 4–6 h – initial phase of adhesion of *S. mutans* to a flat surface of polystyrene; *S. mutans* adheres to the polystyrene surface primarily using a sucrose-dependent mechanism (based on the glucosyltransferase and glucan-binding proteins); 8–14 h – biofilm occurrence, at this stage there is an irreversible merger of the bacteria with the surface and then the formation of the extracellular matrix (ECM) which protects against host defense factors and drying; 16 h – biofilm maturation, during which the matrix is still being formed and other bacterial species also attach to the biofilm, bacteria synthesize extracellular polymers (soluble and insoluble glucans, fructans, and heteropolymers) which are components of the plaque matrix. The appearance of the matrix is a feature of all biofilms, but it is more than a chemical scaffold that maintains the biofilm shape. The matrix is biologically active and retains water, nutrients and enzymes within the biofilm structure: **A** – polymeric ECM, which has an open architecture with nutrient channels, spaces, and other properties, e.g., environmental heterogeneity (pH and oxygen gradients, co-adhesion); **B** – water channels; **C** – *S. mutans* cell. The original magnification: 4000x.

**FIGURE 4:**
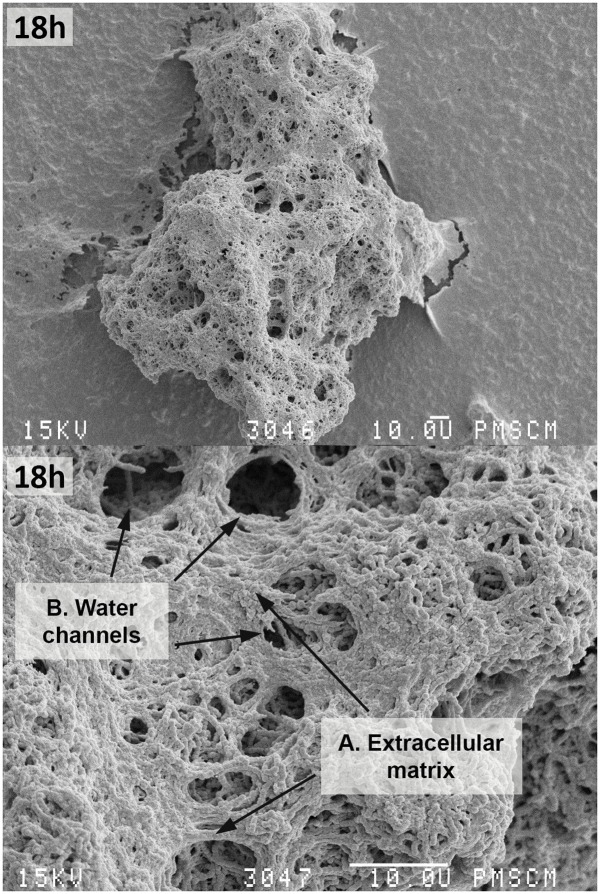
**Scanning electron microscopy of the single-species mature biofilm formed by *S. mutans* cells after 18 h**. The photo shows *S. mutans* cells **(C)** forming a mature biofilm with a visible ECM **(A)** constituting the scaffold of the entire biofilm structure with nutrient channels, **(B)** and other biofilm properties like water channels. The original magnification: 400x, 2000x.

*In vitro* biofilm formation was also analyzed using a SEM, where mature single-species biofilms were observed after 18 h of incubation (**Figures 3**, **4**). **Figures 3**, **4** shows the *S. mutans* cells, extracellular matrix (ECM) and water channels responsible for nutrition within the biofilm. The stages of biofilm formation are clearly identifiable: from the initial phase of the microorganism adhesion to the surface of a flat polystyrene; biofilm maturation when an exopolysaccharide matrix is created and other bacteria species join the biofilm; and finally, the formation of a mature structure and a slowdown in the growth tempo of individual bacteria.

### The Relationship between Pyruvate Kinase Activity and the Amount of Microorganisms (log CFU/ml) and the Biomass of the Formed Biofilm (OD) at Particular Time Points of Single-Species Biofilm Formation

**FIGURE 5:**
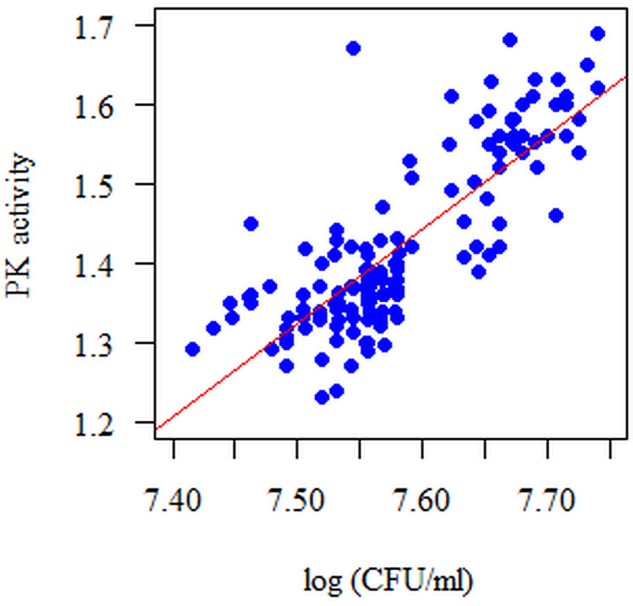
**Diagram of the amount of biofilm-forming microorganisms (*S. mutans*) (log CFU/ml) with respect to PK activity in the 18 h single-species biofilm**. The analyzed features had no normal distribution (*p* < 0.05, Shapiro–Wilk test). The Spearman correlation coefficient was used for the analysis. The correlation between the amount of microorganisms expressed as log CFU/ml and pyruvate kinase activity (PK) is statistically significant at each time point (*p* < 0.05). These dependencies are positive, i.e., the more microorganisms there are (log CFU/ml), the higher the PK activity and *vice versa*.

The correlation between the log (CFU/ml) and PK activity is statistically significant at each time point of single-species biofilm formation (*p* < 0.05). The strongest correlation between the measured parameters was observed after 18 h of biofilm formation, *r* = 0.771, *p* < 0.001; Spearman correlation coefficient (**Figure 5**). These relationships are positive: the higher the log (CFU/ml), the higher the PK activity, and *vice versa*.

**FIGURE 6:**
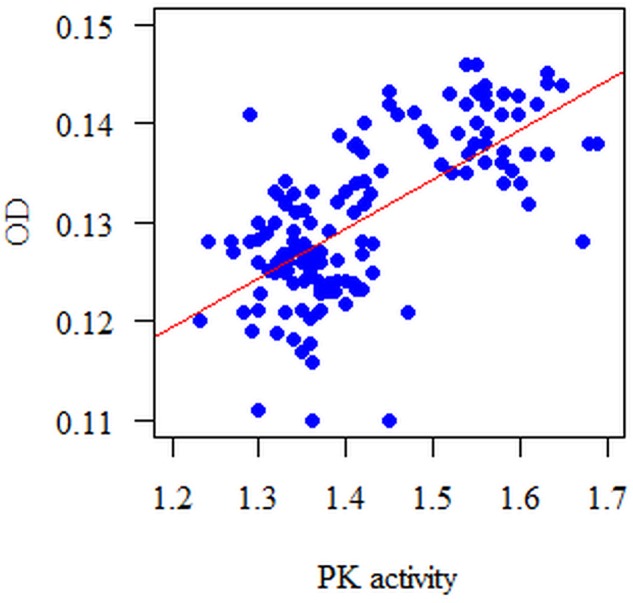
**Diagram of the biofilm biomass (OD = 540 nm) with respect to PK activity in the 18 h single-species biofilm**. The analyzed features had no normal distribution (*p* < 0.05, Shapiro–Wilk test); therefore, the Spearman correlation coefficient was used for the analysis. The correlation between the PK and the biomass of the formed biofilm (OD) is statistically significant at each time point (*p* < 0.05). These dependencies are positive, i.e., the higher the PK activity, the bigger the biomass measured at a wavelength λ_max_ = 540 nm (OD) and *vice versa*.

Similarly, attention has been paid to the relationship between the PK activity at particular time points and the biomass of a formed biofilm (OD = 540 nm). These relationships are positive: the higher the PK activity, the higher the OD, and *vice versa*. This time, correlation between the measured parameters in a mature biofilm (after 18 h) was strong, *r* = 0.771, *p* < 0.001; Spearman correlation coefficient (**Figure 6**).

### Analysis of Pyruvate Kinase Activity with Respect to Caries Severity

At each time point of biofilm formation, the PK activity in the cavitated group was significantly higher than in the non-cavitated group (*p* < 0.05 for all time points; Mann–Whitney Test). Differences at particular time points were as follows: 1.16 ± 1.14–1.21 (median and Q_1_–Q_3_ quartiles) after 4 h for the non-cavitated group (*n* = 97) and 1.24 ± 1.23–1.25 for the cavitated one (*n* = 46). After 6 h: 1.18 ± 1.16–1.23 for the non-cavitated and 1.26 ± 1.24–1.27 for the cavitated group. After 8 h the median values were: 1.2 ± 1.18–1.25 in the non-cavitated and 1.28 ± 1.26–1.29 in the cavitated group. At subsequent points of time, PK activity increased steadily, peaking at 18 h of biofilm formation and equaling 1.36 ± 1.33–1.4 in the non-cavitated group and 1.56 ± 1.52–1.6 in the cavitated one. After 20 h of biofilm formation, PK activity consistently decreased, giving after 24 h median values of 1.26 ± 1.24–1.3 in the non-cavitated and 1.46 ± 1.38–1.48 in the cavitated group (*p* < 0.05, Mann–Whitney test).

### Pyruvate Kinase (PK) Activity in Particular Biotypes

Four analyzed biotypes statistically differed in terms of the activities of PK at each of the time points of biofilm formation (*p* < 0.05, Kruskal–Wallis test). After 4, 6, 8, 10, 12, and 14 h, PK activities in biotypes I and II were significantly higher than in biotype III (*p* = 0.014, *p* = 0.019, *p* = 0.012, *p* = 0.004, *p* = 0.004, and *p* = 0.011, respectively; *post hoc* Dunn’s test, Kruskal–Wallis test). After 16 h, the PK activity in biotype II was significantly higher than in biotype III (*p* = 0.014; *post hoc* Dunn’s test, Kruskal–Wallis test). After 18 h, the activities of PK in biotypes I, II, and IV were significantly higher than in biotype III (*p* = 0.002; *post hoc* Dunn’s test, Kruskal–Wallis test). After 20 and 22 h, the activities of PK in biotypes II and IV were significantly higher than in biotype III (*p* = 0.004 for both time points; *post hoc* Dunn’s test, Kruskal–Wallis test). After 24 h, the PK activity in biotype IV was significantly higher than in biotype III (*p* = 0.013; *post hoc* Dunn’s test, Kruskal–Wallis test) (**Table 1**).

**Table 1:** Pyruvate kinase activity according to biotypes (I–IV) from the arbitrary method.

Time (h)	Biotype	*N*	Mean	*SD*	Median	Minimum	Maximum	Q_1_	Q_3_	*p*^∗^
4	I	62	1.19	0.04	1.2	1.11	1.27	1.14	1.22	*p* = 0.014
	II	23	1.21	0.05	1.24	1.12	1.29	1.17	1.25	
	III	50	1.19	0.05	1.17	1.11	1.29	1.14	1.23	I, II, >III
	IV	8	1.22	0.05	1.24	1.14	1.26	1.19	1.24	
6	I	62	1.21	0.04	1.22	1.13	1.28	1.17	1.24	*p* = 0.019
	II	23	1.23	0.05	1.26	1.13	1.29	1.19	1.27	
	III	50	1.2	0.05	1.19	1.12	1.3	1.16	1.25	I, II, >III
	IV	8	1.23	0.05	1.25	1.15	1.29	1.21	1.27	
8	I	62	1.23	0.04	1.24	1.14	1.3	1.19	1.26	*p* = 0.012
	II	23	1.28	0.16	1.27	1.16	1.98	1.21	1.29	
	III	50	1.22	0.05	1.22	1.14	1.34	1.18	1.26	I, II, >III
	IV	8	1.26	0.05	1.27	1.19	1.33	1.23	1.3	
10	I	62	1.25	0.04	1.25	1.16	1.33	1.21	1.28	*p* = 0.004
	II	23	1.28	0.05	1.3	1.19	1.34	1.23	1.32	
	III	50	1.25	0.05	1.25	1.15	1.37	1.2	1.28	I, II, >III
	IV	8	1.29	0.06	1.31	1.21	1.36	1.25	1.33	
12	I	62	1.27	0.04	1.27	1.18	1.36	1.24	1.31	*p* = 0.004
	II	23	1.3	0.05	1.32	1.21	1.36	1.25	1.34	
	III	50	1.27	0.05	1.26	1.18	1.39	1.23	1.29	I, II, >III
	IV	8	1.31	0.06	1.32	1.24	1.39	1.26	1.35	
14	I	62	1.29	0.05	1.3	1.2	1.39	1.26	1.33	*p* = 0.011
	II	23	1.32	0.05	1.34	1.22	1.38	1.27	1.36	
	III	50	1.29	0.05	1.28	1.21	1.44	1.25	1.32	I, II, >III
	IV	8	1.33	0.06	1.33	1.26	1.42	1.29	1.36	
16	I	62	1.32	0.05	1.32	1.21	1.42	1.28	1.36	*p* = 0.014
	II	23	1.35	0.04	1.36	1.26	1.4	1.33	1.39	
	III	50	1.32	0.06	1.31	1.21	1.46	1.27	1.35	II > III
	IV	8	1.37	0.06	1.36	1.28	1.44	1.32	1.43	
18	I	62	1.41	0.11	1.38	1.23	1.68	1.34	1.46	*p* = 0.002
	II	23	1.48	0.11	1.45	1.33	1.69	1.38	1.56	
	III	50	1.4	0.1	1.37	1.27	1.65	1.33	1.42	I, II, IV>
	IV	8	1.52	0.11	1.54	1.34	1.63	1.47	1.62	III
20	I	62	1.36	0.09	1.34	1.19	1.57	1.3	1.41	*p* = 0.004
	II	23	1.43	0.1	1.41	1.28	1.6	1.34	1.5	
	III	50	1.36	0.1	1.33	1.24	1.57	1.28	1.4	II, IV>
	IV	8	1.47	0.11	1.5	1.27	1.6	1.42	1.54	III
22	I	62	1.34	0.09	1.31	1.16	1.55	1.27	1.38	*p* = 0.004
	II	23	1.39	0.09	1.39	1.25	1.55	1.32	1.47	
	III	50	1.33	0.1	1.29	1.22	1.53	1.26	1.38	II, IV>
	IV	8	1.44	0.1	1.46	1.25	1.54	1.4	1.51	III
24	I	62	1.31	0.08	1.27	1.16	1.53	1.25	1.34	*p* = 0.013
	II	23	1.36	0.1	1.36	1.23	1.54	1.29	1.45	
	III	50	1.31	0.1	1.27	1.2	1.5	1.24	1.36	IV > III
	IV	8	1.4	0.1	1.44	1.22	1.49	1.35	1.48	

*^∗^Kruskal–Wallis test and *post hoc* results (Dunn’s test) were used for the analysis*.

Three analyzed clusters statistically differed in terms of the activities of PK at each of the time points of biofilm formation (*p* < 0.05, Kruskal–Wallis test). At each of the time points, PK activity in cluster A was significantly higher than in the other (B, C) clusters (*p* ≤ 0.001 for all time points; *post hoc* Dunn’s test, Kruskal–Wallis test) (**Table 2**).

**Table 2:** Pyruvate kinase activity according to biotypes (A–C) from the clusterization method.

Time (h)	Cluster	*N*	Mean	*SD*	Median	Minimum	Maximum	Q_1_	Q_3_	*p*^∗^
4	A	12	1.25	0.01	1.25	1.22	1.27	1.24	1.25	*p* < 0.001
	B	71	1.19	0.05	1.19	1.11	1.29	1.15	1.22	
	C	60	1.19	0.05	1.18	1.11	1.29	1.15	1.23	A > BC
6	A	12	1.26	0.02	1.27	1.24	1.29	1.26	1.27	*p* < 0.001
	B	71	1.21	0.05	1.22	1.13	1.29	1.16	1.25	
	C	60	1.21	0.05	1.21	1.12	1.3	1.16	1.25	A > BC
8	A	12	1.28	0.02	1.28	1.25	1.32	1.27	1.29	*p* < 0.001
	B	71	1.24	0.1	1.24	1.14	1.98	1.19	1.26	
	C	60	1.23	0.05	1.23	1.14	1.34	1.19	1.26	A > BC
10	A	12	1.31	0.02	1.31	1.27	1.36	1.3	1.32	*p* < 0.001
	B	71	1.25	0.05	1.25	1.16	1.34	1.21	1.29	
	C	60	1.25	0.05	1.25	1.15	1.37	1.21	1.29	A > BC
12	A	12	1.34	0.02	1.34	1.3	1.39	1.32	1.34	*p* < 0.001
	B	71	1.27	0.05	1.27	1.18	1.36	1.24	1.31	
	C	60	1.27	0.05	1.26	1.18	1.39	1.24	1.3	A > BC
14	A	12	1.35	0.03	1.35	1.32	1.41	1.33	1.36	*p* = 0.001
	B	71	1.29	0.05	1.3	1.2	1.39	1.26	1.34	
	C	60	1.29	0.05	1.28	1.21	1.44	1.25	1.33	A > BC
16	A	12	1.38	0.03	1.37	1.34	1.44	1.35	1.39	*p* = 0.003
	B	71	1.32	0.05	1.32	1.21	1.42	1.28	1.37	
	C	60	1.32	0.06	1.31	1.21	1.46	1.28	1.35	A > BC
18	A	12	1.54	0.1	1.57	1.36	1.69	1.47	1.62	*p* = 0.001
	B	71	1.41	0.1	1.38	1.23	1.67	1.34	1.46	
	C	60	1.41	0.11	1.37	1.27	1.68	1.33	1.48	A > BC
20	A	12	1.5	0.08	1.52	1.3	1.6	1.48	1.55	*p* = 0.001
	B	71	1.37	0.09	1.35	1.19	1.56	1.3	1.41	
	C	60	1.37	0.1	1.33	1.24	1.6	1.28	1.45	A > BC
22	A	12	1.47	0.07	1.49	1.29	1.55	1.45	1.51	*p* < 0.001
	B	71	1.34	0.09	1.32	1.16	1.55	1.27	1.38	
	C	60	1.34	0.1	1.29	1.22	1.54	1.26	1.42	A > BC
24	A	12	1.45	0.06	1.46	1.28	1.54	1.44	1.48	*p* < 0.001
	B	71	1.31	0.08	1.29	1.16	1.53	1.25	1.35	
	C	60	1.32	0.1	1.27	1.2	1.5	1.24	1.38	A > BC

*^∗^Kruskal–Wallis test and *post hoc* results (Dunn’s test) were used for the analysis*.

### The Amount of Microorganisms (log CFU/ml) in Particular Biotypes

The analyzed biotypes significantly differed in terms of the amounts of microorganisms forming a biofilm CFU/ml for eight time points (*p* < 0.05, Kruskal–Wallis test). *Post hoc* analysis showed that the number of microorganisms contained in the biofilm after 18 h in cluster A was significantly higher than in clusters B and C (in terms of OD and CFU differentiation, division into three clusters is better) (*p* = 0.006; *post hoc* Dunn’s test). Taking into account the differences in log CFU/ml between four biotypes (from arbitrary method), a great diversity was observed. *Post hoc* analysis (Kruskal–Wallis test + Dunn’s test; *p* < 0.05) showed that after 4 and 6 h CFU/ml in biotype IV was significantly higher than in biotype I (*p* = 0.284 and *p* = 0.081; *post hoc* Dunn’s test); after 14 h, CFU/ml in biotype IV was significantly higher than in biotype III (*p* = 0.049; *post hoc* Dunn’s test); after 16 h, CFU/ml in biotype IV was significantly higher than in biotypes I and III (*p* = 0.026; *post hoc* Dunn’s test); after 18 and 20 h, CFU/ml in biotype II was significantly higher than in biotype III (*p* = 0.017; *post hoc* Dunn’s test); and after 20 and 24 h, CFU/ml in biotype II was significantly higher than in biotypes III and I (*p* = 0.037 and *p* = 0.022; *post hoc* Dunn’s test) (**Tables 3**, **4**).

**Table 3:** Amounts of microorganisms forming a biofilm (log CFU/ml) according to biotypes (I–IV) from the arbitrary method.

Time (h)	Biotype	*N*	Mean	*SD*	Median	Minimum	Maximum	Q1	Q3	*p*^∗^
4	I	62	7.09	0.07	7.08	7	7.32	7.04	7.11	*p* = 0.284
	II	23	7.12	0.08	7.08	7	7.32	7.08	7.15	
	III	50	7.09	0.06	7.08	7	7.28	7.04	7.11	
	IV	8	7.13	0.11	7.1	7	7.28	7.04	7.24	
6	I	62	7.14	0.07	7.11	7.04	7.36	7.11	7.18	*p* = 0.081
	II	23	7.18	0.08	7.15	7.08	7.38	7.11	7.23	
	III	50	7.15	0.06	7.15	7.08	7.32	7.11	7.18	
	IV	8	7.21	0.12	7.18	7.08	7.34	7.11	7.34	
8	I	62	7.2	0.07	7.18	7.08	7.4	7.15	7.22	*p* = 0.025
	II	23	7.23	0.07	7.2	7.15	7.38	7.18	7.26	
	III	50	7.21	0.07	7.2	7.11	7.38	7.18	7.23	IV > I
	IV	8	7.27	0.1	7.24	7.15	7.4	7.22	7.38	
10	I	62	7.25	0.07	7.23	7.15	7.45	7.2	7.28	*p* = 0.034
	II	23	7.28	0.08	7.26	7.18	7.41	7.2	7.34	
	III	50	7.26	0.07	7.26	7.15	7.41	7.21	7.28	IV > I
	IV	8	7.32	0.09	7.28	7.23	7.43	7.27	7.42	
12	I	62	7.31	0.07	7.32	7.18	7.52	7.26	7.34	*p* = 0.067
	II	23	7.33	0.07	7.34	7.23	7.45	7.27	7.38	
	III	50	7.32	0.07	7.32	7.2	7.46	7.26	7.36	
	IV	8	7.38	0.06	7.36	7.3	7.46	7.34	7.45	
14	I	62	7.37	0.07	7.38	7.23	7.56	7.34	7.4	*p* = 0.049
	II	23	7.4	0.07	7.4	7.26	7.52	7.36	7.45	
	III	50	7.37	0.07	7.36	7.23	7.52	7.34	7.41	IV > III
	IV	8	7.44	0.07	7.41	7.36	7.52	7.39	7.51	
16	I	62	7.43	0.07	7.41	7.32	7.72	7.38	7.45	*p* = 0.026
	II	23	7.46	0.08	7.45	7.34	7.67	7.4	7.51	
	III	50	7.43	0.07	7.41	7.3	7.67	7.38	7.46	IV > III.I
	IV	8	7.49	0.07	7.48	7.4	7.57	7.44	7.55	
18	I	62	7.57	0.07	7.56	7.43	7.71	7.53	7.63	*p* = 0.017
	II	23	7.61	0.07	7.64	7.51	7.74	7.56	7.66	
	III	50	7.57	0.08	7.56	7.41	7.74	7.53	7.59	II > III
	IV	8	7.64	0.08	7.67	7.48	7.72	7.62	7.69	
20	I	62	7.49	0.08	7.45	7.36	7.66	7.43	7.54	*p* = 0.037
	II	23	7.53	0.08	7.56	7.34	7.64	7.5	7.58	
	III	50	7.49	0.09	7.45	7.36	7.67	7.41	7.55	II > III
	IV	8	7.54	0.09	7.53	7.41	7.65	7.47	7.62	
22	I	62	7.45	0.08	7.41	7.32	7.65	7.4	7.5	*p* = 0.015
	II	23	7.5	0.08	7.52	7.3	7.66	7.46	7.55	
	III	50	7.45	0.09	7.41	7.32	7.65	7.38	7.5	II > III.I
	IV	8	7.48	0.1	7.45	7.34	7.63	7.41	7.56	
24	I	62	7.4	0.08	7.38	7.26	7.59	7.35	7.44	*p* = 0.022
	II	23	7.45	0.07	7.45	7.28	7.6	7.43	7.51	
	III	50	7.4	0.09	7.38	7.26	7.61	7.34	7.45	II > III.I
	IV	8	7.45	0.1	7.41	7.32	7.59	7.38	7.54	

*^∗^Kruskal–Wallis test and *post hoc* results (Dunn’s test) were used for the analysis*.

**Table 4:** Amounts of microorganisms forming a biofilm (log CFU/ml) according to biotypes (A–C) from the clusterization method.

Time (h)	Cluster	*N*	Mean	*SD*	Median	Minimum	Maximum	Q1	Q3	*p*^∗^
4	A	12	7.16	0.08	7.15	7.08	7.28	7.08	7.24	*p* = 0.009
	B	71	7.09	0.08	7.08	7	7.32	7.04	7.11	
	C	60	7.09	0.06	7.08	7	7.28	7.04	7.11	A > BC
6	A	12	7.22	0.1	7.2	7.08	7.34	7.15	7.31	*p* = 0.037
	B	71	7.15	0.07	7.11	7.08	7.38	7.11	7.18	
	C	60	7.15	0.06	7.15	7.04	7.34	7.11	7.18	A > BC
8	A	12	7.27	0.09	7.26	7.15	7.4	7.2	7.36	*p* = 0.021
	B	71	7.2	0.07	7.18	7.11	7.4	7.15	7.23	
	C	60	7.21	0.07	7.2	7.08	7.38	7.18	7.23	A > BC
10	A	12	7.32	0.08	7.34	7.2	7.43	7.25	7.4	*p* = 0.031
	B	71	7.26	0.07	7.23	7.15	7.45	7.2	7.28	
	C	60	7.26	0.07	7.26	7.15	7.43	7.2	7.28	A > BC
12	A	12	7.37	0.07	7.38	7.26	7.45	7.3	7.43	*p* = 0.105
	B	71	7.32	0.07	7.32	7.2	7.52	7.26	7.36	
	C	60	7.32	0.07	7.32	7.18	7.46	7.28	7.36	
14	A	12	7.43	0.07	7.44	7.32	7.52	7.38	7.48	*p* = 0.055
	B	71	7.38	0.07	7.38	7.26	7.56	7.34	7.41	
	C	60	7.38	0.07	7.37	7.23	7.52	7.34	7.41	
16	A	12	7.48	0.07	7.51	7.38	7.57	7.41	7.53	*p* = 0.089
	B	71	7.43	0.08	7.41	7.32	7.72	7.38	7.45	
	C	60	7.43	0.07	7.41	7.3	7.67	7.38	7.48	
18	A	12	7.65	0.05	7.65	7.56	7.74	7.64	7.68	*p* = 0.006
	B	71	7.57	0.07	7.56	7.43	7.72	7.53	7.64	
	C	60	7.58	0.08	7.57	7.41	7.74	7.53	7.65	A > BC
20	A	12	7.57	0.04	7.57	7.51	7.65	7.54	7.6	*p* = 0.005
	B	71	7.49	0.08	7.46	7.34	7.66	7.43	7.55	
	C	60	7.49	0.09	7.46	7.36	7.67	7.41	7.56	A > BC
22	A	12	7.54	0.04	7.53	7.48	7.63	7.52	7.56	*p* = 0.001
	B	71	7.45	0.08	7.43	7.3	7.66	7.4	7.51	
	C	60	7.45	0.09	7.41	7.32	7.65	7.38	7.52	A > BC
24	A	12	7.49	0.05	7.5	7.45	7.59	7.45	7.52	*p* = 0.001
	B	71	7.41	0.08	7.4	7.26	7.6	7.36	7.45	
	C	60	7.4	0.09	7.38	7.26	7.61	7.34	7.45	A > BC

*^∗^Kruskal–Wallis test and *post hoc* results (Dunn’s test) were used for the analysis*.

### Biofilm Formation by Determining Total Biomass [Optical Density (OD)] in Particular Biotypes

The analyzed biotypes were significantly different in terms of the biomass of a formed biofilm (OD) for eight time points (*p* < 0.05, Kruskal–Wallis test). *Post hoc* analysis revealed that the biofilm biomass after 18 h in cluster A was significantly higher than in clusters B and C (*p* < 0.05, Dunn’s test).

Taking into account the differences in OD between the four biotypes (from the arbitrary method) a great diversity can be observed. *Post hoc* analysis (Kruskal–Wallis test + Dunn’s test; *p* < 0.05) showed that after 6 h, OD in biotype II was significantly higher than in biotype I; after 8, 10, 14, 18, and 20 h, OD in biotype II was significantly higher than in biotypes I and III; after 16 and 22 h, OD in biotype II was significantly higher than in biotype III.

Considering the division into three clusters (clusterization method), these results were more consistent than those from the arbitrary method. Adoption of the above-mentioned criterion in completing the division into three clusters seems to be more reasonable (in the context of OD and CFU/ml differentiation), as in that case the same relationship can be observed at each time point of biofilm formation; while for four biotypes (arbitrary method) this relationship is quite strongly “mixed” (**Tables 5**, **6**).

**Table 5:** Biofilm formation by determining total biomass [optical density (OD)] according to biotypes (I–IV) from the arbitrary method.

Time (h)	Biotype	*N*	Mean	*SD*	Median	Minimum	Maximum	Q1	Q3	*p*^∗^
4	I	62	0.03	0.01	0.03	0.02	0.04	0.03	0.04	*p* = 0.077
	II	23	0.04	0.01	0.04	0.03	0.04	0.03	0.04	
	III	50	0.03	0.01	0.03	0.02	0.04	0.03	0.04	
	IV	8	0.03	0.01	0.04	0.02	0.04	0.03	0.04	
6	I	62	0.04	0.01	0.04	0.03	0.06	0.04	0.05	*p* = 0.036
	II	23	0.05	0.01	0.05	0.04	0.06	0.04	0.05	
	III	50	0.04	0.01	0.04	0.03	0.06	0.04	0.05	II > I
	IV	8	0.05	0.01	0.05	0.03	0.06	0.04	0.05	
8	I	62	0.05	0.01	0.05	0.04	0.07	0.05	0.06	*p* = 0.035
	II	23	0.06	0.01	0.06	0.05	0.07	0.05	0.06	
	III	50	0.05	0.01	0.05	0.04	0.07	0.05	0.06	II > III.I
	IV	8	0.06	0.01	0.06	0.04	0.07	0.06	0.07	
12	I	62	0.06	0.01	0.06	0.05	0.08	0.06	0.07	*p* = 0.008
	II	23	0.07	0.01	0.07	0.05	0.08	0.06	0.07	
	III	50	0.06	0.01	0.06	0.05	0.08	0.06	0.07	II > III.I
	IV	8	0.07	0.01	0.07	0.06	0.08	0.07	0.08	
14	I	62	0.07	0.01	0.07	0.06	0.09	0.07	0.08	*p* = 0.019
	II	23	0.08	0.01	0.08	0.07	0.09	0.07	0.08	
	III	50	0.07	0.01	0.07	0.06	0.09	0.07	0.08	II > III.I
	IV	8	0.08	0.01	0.08	0.06	0.08	0.08	0.08	
16	I	62	0.09	0.01	0.09	0.06	0.11	0.08	0.1	*p* = 0.02
	II	23	0.1	0.01	0.1	0.08	0.11	0.09	0.1	
	III	50	0.09	0.01	0.09	0.07	0.11	0.08	0.1	II > III
	IV	8	0.1	0.01	0.1	0.08	0.11	0.09	0.1	
18	I	62	0.13	0.01	0.13	0.11	0.15	0.12	0.14	*p* = 0.001
	II	23	0.14	0.01	0.14	0.12	0.14	0.13	0.14	
	III	50	0.13	0.01	0.13	0.11	0.14	0.12	0.13	II > III.I
	IV	8	0.14	0.01	0.14	0.12	0.15	0.13	0.14	
20	I	62	0.12	0.01	0.12	0.1	0.14	0.12	0.13	*p* = 0.005
	II	23	0.13	0.01	0.13	0.12	0.13	0.12	0.13	
	III	50	0.12	0.01	0.12	0.1	0.13	0.12	0.12	II > III.I
	IV	8	0.12	0.01	0.13	0.11	0.13	0.12	0.13	
22	I	62	0.11	0.01	0.11	0.09	0.13	0.11	0.12	*p* = 0.033
	II	23	0.12	0	0.12	0.11	0.12	0.12	0.12	
	III	50	0.11	0.01	0.11	0.09	0.12	0.11	0.12	II > III
	IV	8	0.12	0.01	0.12	0.11	0.13	0.11	0.12	
24	I	62	0.1	0.01	0.11	0.08	0.12	0.1	0.11	*p* = 0.071
	II	23	0.11	0.01	0.11	0.1	0.12	0.11	0.11	
	III	50	0.1	0.01	0.1	0.08	0.12	0.09	0.11	
	IV	8	0.11	0.01	0.11	0.09	0.11	0.1	0.11	

*^∗^Kruskal–Wallis test and *post hoc* results (Dunn’s test) were used for the analysis*.

**Table 6:** Biofilm formation by determining total biomass [optical density (OD)] according to biotypes (A–C) from the clusterization method.

Time (h)	Cluster	*N*	Mean	*SD*	Median	Minimum	Maximum	Q1	Q3	*p*^∗^
4	A	12	0.04	0	0.04	0.03	0.04	0.04	0.04	*p* = 0.007
	B	71	0.03	0.01	0.03	0.02	0.04	0.03	0.04	
	C	60	0.03	0.01	0.03	0.02	0.04	0.03	0.04	A > BC
6	A	12	0.05	0.01	0.05	0.04	0.06	0.05	0.05	*p* = 0.006
	B	71	0.04	0.01	0.04	0.03	0.06	0.04	0.05	
	C	60	0.04	0.01	0.04	0.03	0.06	0.04	0.05	A > BC
8	A	12	0.06	0.01	0.06	0.05	0.07	0.06	0.06	*p* = 0.022
	B	71	0.05	0.01	0.05	0.04	0.07	0.05	0.06	
	C	60	0.05	0.01	0.05	0.04	0.07	0.05	0.06	A > BC
12	A	12	0.07	0	0.07	0.06	0.08	0.07	0.08	*p* = 0.008
	B	71	0.06	0.01	0.06	0.05	0.08	0.06	0.07	
	C	60	0.06	0.01	0.06	0.05	0.08	0.06	0.07	A > BC
14	A	12	0.08	0	0.08	0.07	0.09	0.08	0.08	*p* = 0.017
	B	71	0.07	0.01	0.07	0.06	0.09	0.07	0.08	
	C	60	0.07	0.01	0.07	0.06	0.09	0.07	0.08	A > BC
16	A	12	0.1	0.01	0.1	0.08	0.1	0.1	0.1	*p* = 0.029
	B	71	0.09	0.01	0.09	0.06	0.11	0.08	0.1	
	C	60	0.09	0.01	0.09	0.07	0.11	0.08	0.1	A > BC
18	A	12	0.14	0	0.14	0.13	0.14	0.13	0.14	*p* = 0.006
	B	71	0.13	0.01	0.13	0.11	0.15	0.12	0.14	
	C	60	0.13	0.01	0.13	0.11	0.15	0.12	0.14	A > BC
20	A	12	0.13	0	0.13	0.12	0.13	0.13	0.13	*p* = 0.032
	B	71	0.12	0.01	0.12	0.1	0.14	0.12	0.13	
	C	60	0.12	0.01	0.12	0.1	0.13	0.12	0.13	A > BC
22	A	12	0.12	0	0.12	0.11	0.12	0.12	0.12	*p* = 0.093
	B	71	0.11	0.01	0.11	0.09	0.13	0.11	0.12	
	C	60	0.11	0.01	0.12	0.09	0.13	0.11	0.12	
24	A	12	0.11	0.01	0.11	0.1	0.12	0.11	0.11	*p* = 0.298
	B	71	0.1	0.01	0.1	0.08	0.12	0.1	0.11	
	C	60	0.1	0.01	0.1	0.08	0.12	0.1	0.11	

*^∗^Kruskal–Wallis test and *post hoc* results (Dunn’s test) were used for the analysis*.

## Discussion

As an etiological factor of dental caries, *S. mutans* has a number of features, such as the capacity for adhesion and biofilm formation, which determine its pathogenicity. Under oral cavity conditions, *S. mutans* is characterized by altered metabolism compared to cells of physiological flora. This is based on glycolysis also occurring in the presence of oxygen (Pasteur effect). Hypoxia (oxygen concentration <2%) which prevails within the biofilm, increases the expression of genes encoding glycolytic enzymes and inhibits oxidative phosphorylation. Glycolysis is a major source of energy and building blocks associated with sugar metabolism in eukaryotic cells. The role of glycolysis is dual: degradation of sugars with ATP formation; and the supply of necessary nutrient substrates for other biochemical reactions. The conversion of glucose to pyruvate in eukaryotic cells is essential for vital cell functions. During glycolysis, PK catalyzes irreversible reactions and is one of the three enzymes (along with hexokinase and phosphofructokinase PFK) which regulate the pace of the entire process.

In cariogenic bacterial cells, PK is responsible for the homeostasis of redox reactions, as evidenced by the activation of the pentose pathway which reduces the accumulation of reactive oxygen species and protects bacteria against oxidative stress, thus facilitating their growth (Jurczak et al., 2017).

The biotypes we obtained are in line with available literature; the frequencies of individual biotypes characterized by given enzyme activity profiles are comparable to those of the corresponding biotypes obtained in Facklam et al. (1974) and Yoo et al. (2007).

A comparison of our results with those obtained by Yoo et al. (2007) shows that from 143 strains 50 showed the same profile: positive reactions for all compounds except arginine, the remaining four strains differed in terms of a lack of melibiose fermentation. Due to the lack of difference between the strains studied in the compounds’ metabolism by Yoo et al. (2007) (with the exception of melibiose), our proposed scheme based on the metabolism of inulin, melibiose, and tagatose is more appropriate for establishing *S. mutans* biotypes in the Polish population. The presented method enables better differentiation of the species. Using the scheme introduced by Shklair and Keene (1974), based on biotyping by Yoo et al. (2007), would have enabled only two biotypes to have been obtained in our study; hence, the biotypes we have established seem to be more adequate. In addition, the number and type of biotypes obtained by Yoo et al. (2007) do not transfer into our results, because in the isolated *S. mutans* strains, three (mannitol, sorbitol, raffinose) of the four enzymes were not significantly different in their activity.

Facklam identified biotypes for clinical *S. mutans* strains isolated from both dental plaque (50) and blood (54), where the frequencies of individual enzymes are comparable to that observed in our study: mannose, trehalose, and esculin over 95%; raffinose and sorbitol over 85%. In the case of melibiose fermentation, 83.22% of the strains we tested had the activity of the corresponding enzyme, whereas Facklam observed this feature in less than half of the isolates from plaque (43%) and 88% of strains isolated from blood (1974).

Other teams, e.g., De La Higuera et al. (1999), used different biotyping criteria, taking into account distribution based on the activity of phosphatase, valine-aryl-amidase and α-galactosidase. However, as in the De La Higuera’s study, phosphatase from our isolated *S. mutans* strains was characterized by variable activity (11.89%). However, because of the qualification criteria of enzymes included in the biotyping of 15–85%, this feature was rejected in biotyping.

Another way to determine biotypes is the use of the statistical methods of clusterization. This allows researchers to take into account the activity of all enzymes from the STREPTOtest 24 in defining the biotype. It appears that the best differentiation of biotypes can be achieved on the basis of the activity of the three enzymes degrading inulin (INU), melibiose (MLB), and tagatose (TGT). It is striking why *Streptococcus* strains differ in terms of the activity of enzymes responsible for the hydrolysis of melibiose within the group of children affected by caries (**Figure 3** cluster A is only present in the cavitated group; biotype II is characterized by a lack of hydrolysis of inulin, degrades tagatose and clearly differs in melibiose hydrolysis). Variability associated with the distribution of melibiose in strains from children with advanced caries compared with children from the non-cavitated group was noticed in both arbitrary and clusterization methods. When divided into four biotypes (arbitrary method), 11 strains from among those not fermenting melibiose were in the ‘red’ cluster, 3 in ‘green’ and 10 in ‘blue.’ When divided into three clusters (clusterization method), 11 strains were in the ‘red’ cluster, 3 in ‘green’ and 10 in ‘blue.’ So, the results of the two biotyping methods were consistent with each other. Among strains with inactive melibiose hydrolysis, a strong variation occurred in the activity of TGT and MLT (50% active and 50% inactive) and bGA, aGA, PHS, INU, SOR, RIB, and RAF (40–60% active). Only when we take the range of 45–55%, do six enzymes remain, giving seven profiles. When these are combined with profiles differing in the activity of one enzyme, there are ultimately four biotypes. Finally, narrow criteria (45–55% range) were adopted to derive a reasonable number of biotypes, which confirms the earlier observation that strains which do not ferment melibiose are highly diverse.

The pathogenicity of certain microbial species such as *S. mutans* is inherent to their capacity for biofilm formation on solid surfaces, e.g., tissues, catheters, or implants (Huang et al., 2013; Cukkemane et al., 2015). This feature allows the microorganisms to create a three-dimensional structure in which cells become more resistant to antibiotics and the external environment, e.g., due to changes as a result of interbacterial interactions and by the presence of the exopolysaccharide matrix that protects the entire structure (Welin-Neilands and Svensäter, 2007; Bowen and Koo, 2011). There are some studies suggesting that *S. mutans* isolates have a greater ability to form biofilm than the isolates of other species of the *Streptococcus* genus which inhabit the human oral cavity environment (Tamura et al., 2009).

The Analyzed biotypes differed significantly in terms of the amount of microorganisms in the biofilm CFU at individual time points (143 analyzed strains). *Post hoc* analysis showed that after 18 h, CFU of biotype II was significantly higher than in biotype III. The clusterization method gave similar observations, in which it was noted that after 18 h, CFU in the biofilm of cluster A was significantly higher than that in clusters B and C. Similar observations were made for the biofilm biomass. Analyzed biotypes differed significantly in terms of biomass (OD) at the individual time points. *Post hoc* analysis showed that after 18 h, OD in biotype II was significantly higher than in biotype III. The clustering method gave similar observations: after 18 h, OD of cluster A was significantly higher than that in clusters B and C.

The obtained biotypes proved to be useful in the differentiation of strains in terms of the activity of PK, which showed a downward trend between clusters A > B > C. Analyzed biotypes were significantly different in terms of PK activity at each time point. *Post hoc* analysis showed that after 4, 6, 8, 14, 18, and 20 h, the PK activity of cluster A was significantly higher than that in cluster D; after 10, 12, 16, 22, and 24 h, the activity of PK biotypes/clusters A and B was significantly higher than that in cluster D (Shapiro–Wilk test *p* < 0.05; *post hoc* Dunn’s test).

Despite the availability of genetic methods allowing better species differentiation, e.g., for epidemiological purposes (Moser et al., 2010), typing on a biochemical level is a potential competitive method due to its low cost. The obtained biotypes allow for a better differentiation of the *S. mutans* species, and thus can facilitate treatment and contribute to our knowledge of the transmission mechanisms of pathogenic bacteria colonizing the human oral cavity.

In this paper, biofilm biomass was evaluated using the CV staining method. Although there are plenty of complex and technology advanced methods (De Leon, 2009; Zijnge et al., 2010; Pantanella et al., 2013) available to study biofilms are complex and technologically advanced, the CV method seems to meet our expectations.

Crystal violet analysis has been proved to be coherent with the results obtained by other methods, i.e., XTT (*r* = 0.76, *p* < 0.05), flatbed scanner (*R^2^* = 0.88), measurement of biofilm thickness (*R^2^* = 0.89), ACC (automated cell counter) and BST (biofilm suspension turbidity) (Peeters et al., 2008; Alnnasouri et al., 2011; Heidrich et al., 2011; Alnuaimi et al., 2013). Both our study and other available literature confirm that there are no significant differences between the results obtained using CV method and CFU/ml counting (Silva et al., 2013; Quishida et al., 2016; Wang et al., 2016).

Our study highlighted the existence of a strong relationship between biofilm formation ability of *S. mutans* and the activity of PK, especially after 18 h of biofilm formation, when the exopolysaccharide matrix is mature and water channels are visible in the already established structure of the biofilm (*r* = 0.771, *p* < 0.001) (**Figures 4–6**). These results are consistent with the results of McNeill and Hamilton (2004), who drew attention to the maximal activity of glycolysis proteins within 24 h of the formation of biofilm. With a decrease in biofilm biomass and a gradual degradation of the cells forming the biofilm there is a decrease in the activity of glycolytic enzymes such as PK. This decrease is associated with limited diffusion of glucose in structurally heterogeneous locations, ultimately reducing the absorption of glucose.

In our studies, we pay attention to the increasing activity of PK depending on the severity of dental caries (*p* < 0.05; Mann–Whitney test) and the growth of the biofilm. This is because, depending on age, composition of biological plaque and the concentration of carbohydrates in the diet, *in vivo* intake of sugar can lead to fluctuations in the pH of human plaque from 7 to 4 in a very short time, as in patients with advanced caries (Stephan, 1944; Yamada et al., 1980). Thus, caries-associated microorganisms not only contribute to the acidification of biofilm, but unlike other bacteria are also able to continue metabolism in low pH due to acid tolerance resistance mechanisms (ATR) (Svensäter et al., 1997, 2001; Welin et al., 2003). With a decrease in environmental pH, the activity of the H^+^/ATPase pump associated with the ATR increases, thus enhancing synthesis of glycolytic enzymes, including glucokinase, aldolase, PK and phosphoglyceromutase. The increased amount of ATP produced during glycolysis and the consequent increased removal of protons from the cell by the H^+^/ATPase maintain the intracellular pH homeostasis. This is also supported by the conversion of l-malate to l-lactate, which further reduces the acidity of the cytoplasm (Dashper and Reynolds, 1996; Wilkins et al., 2002; Lemme et al., 2010).

In our study, a proportionate increase in the activity of to the formed biofilm biomass and the number of CFU/ml can be observed, illustrating their mutual connection and at the same time this may serve as a therapeutic point for potential compounds blocking biofilm formation by inhibiting the metabolic activity of selected glycolytic enzymes. Such an approach could minimize the cariogenic potential of bacteria causing caries and could be an opportunity for a more effective future prevention, possibly also in other therapeutic fields. This could additionally provide an impetus and guide for research on other diseases caused by bacteria.

## Author Contributions

WK was responsible for the concept of the study, and participated in its design. AJ participated in the clinical experiment. WK and PV prepared and analyzed samples, and performed all experiments during the study. WK also performed the statistical analysis and data interpretation. WK was responsible for the manuscript preparation. DK participated in the study design and performed the clinical experiment. MP participated in the study design. AS participated in the study design. KZ-Ś participated in the study design, WK, AS was additionally responsible for corrections leading to the final version of the manuscript. All the authors read and approved the version of the manuscript submitted to the journal.

## Conflict of Interest Statement

The authors declare that the research was conducted in the absence of any commercial or financial relationships that could be construed as a potential conflict of interest.
